# Hemophagocytic lymphohistiocytosis/cytokine release syndrome secondary to neoadjuvant pembrolizumab for triple-negative breast cancer: a case study

**DOI:** 10.3389/fonc.2024.1394543

**Published:** 2024-06-11

**Authors:** Laura Patton, Bethany Monteith, Paul Heffernan, Thomas Herzinger, Brooke E. Wilson

**Affiliations:** ^1^ Department of Oncology, Queen’s University, Kingston, ON, Canada; ^2^ Department of Haematology, Queen’s University, Kingston, ON, Canada; ^3^ Department of Critical Care Medicine, Queen’s University, Kingston, ON, Canada; ^4^ Division of Dermatology, Department of Medicine, Queen’s University, Kingston, ON, Canada; ^5^ Division of Cancer Care and Epidemiology, Queen’s Cancer Research Institute, Kingston, ON, Canada

**Keywords:** breast cancer, immunotherapy, toxicity, cytokine release syndrome, hemophagocityc lymphohistiocytosis

## Abstract

As indications for immune checkpoint inhibitors for breast cancer continue to expand, rare toxicities will emerge that require careful consideration and multidisciplinary management. We report the case of a 40-year-old female receiving neoadjuvant pembrolizumab and chemotherapy for locally advanced triple-negative breast cancer who developed cytokine release syndrome (CRS)/hemophagocytic lymphohistiocytosis (HLH). CRS/HLH secondary to pembrolizumab are scarcely documented in the literature and, to our knowledge, have never been reported in the context of neoadjuvant treatment for breast cancer.

## Introduction

Triple-negative breast cancer (TNBC) is an aggressive malignancy associated with poor prognosis and high risk of early relapse (1). TNBCs are heavily infiltrated by immune cells (2), and a high tumor-infiltrating lymphocyte count has been associated with improved survival (3–5), providing a biological rationale for the use of immune checkpoint blockade in TNBC. Pembrolizumab is a monoclonal antibody that blocks programmed death (PD)-1, resulting in improved anti-tumour activity by tumor-infiltrating T lymphocytes. The practice changing phase III Keynote-522 trial investigated the addition of pembrolizumab to neoadjuvant chemotherapy for patients with early stage TNBC and found improved pathologic complete response rates (pCR 64.8% vs. 51.2%) and event-free survival (84.5% vs. 76.8% at 36 months) when compared to chemotherapy alone (6–8). Pembrolizumab in combination with chemotherapy has also become standard of care for patients with metastatic TNBC and combined positive score >10 based on the Keynote-355 data (9).

As the number of patients treated with immune checkpoint inhibitors (ICIs) in the curative and palliative setting for TNBCs grows, rare toxicities will emerge that require careful consideration and multidisciplinary management. Cytokine release syndrome (CRS) and hemophagocytic lymphohistiocytosis (HLH) secondary to pembrolizumab are scarcely documented in the literature and, to our knowledge, have never been reported in the context of neoadjuvant treatment for breast cancer. HLH is a hyperinflammatory syndrome that occurs due to an overactivated immune response (10). While the primary form of this disease occurs in children, secondary HLH can occur in the context of cancer, infection or autoinflammatory disorders (10). CRS is an excessive systemic immune response characterized by the release of cytokines such as interleukin-6 (IL-6), interferon gamma, tumor necrosis factor alpha, IL-2, and IL-10 by large numbers of activated lymphocytes (11). We present the case of a 40-year-old patient who developed HLH/CRS after exposure to pembrolizumab in the neoadjuvant setting for TNBC.

## Case report

A 40-year patient initially presented to her family physician with left axillary tenderness. A mammogram demonstrated a solid mass with irregular margins in her left breast with multiple left axillary lymph nodes, and biopsy confirmed TNBC. She underwent further staging imaging with a bone scan, computed tomography (CT) neck, chest, abdomen and pelvis and magnetic resonance imaging (MRI) breast. Imaging demonstrated locally advanced disease with nodal involvement but no distant metastases, and she was staged as cT2N2. Multidisciplinary cancer conference consensus was to proceed with curative intent treatment, and she was initiated on the Keynote-522 regimen.

She completed four cycles of treatment with carboplatin, paclitaxel, and pembrolizumab. She had a single episode of non-neutropenic fever, a mild intermittent localized rash affecting the face treated with topical corticosteroid, and a rhinovirus infection resulting in a 1-week delay. After cycle 4, she developed a right eye chemosis and was seen urgently by ophthalmology. This was deemed to be unrelated to immunotherapy and was treated with lubricating eye drops. She then proceeded with the first cycle of doxorubicin, cyclophosphamide, and pembrolizumab.

**Figure 1:**
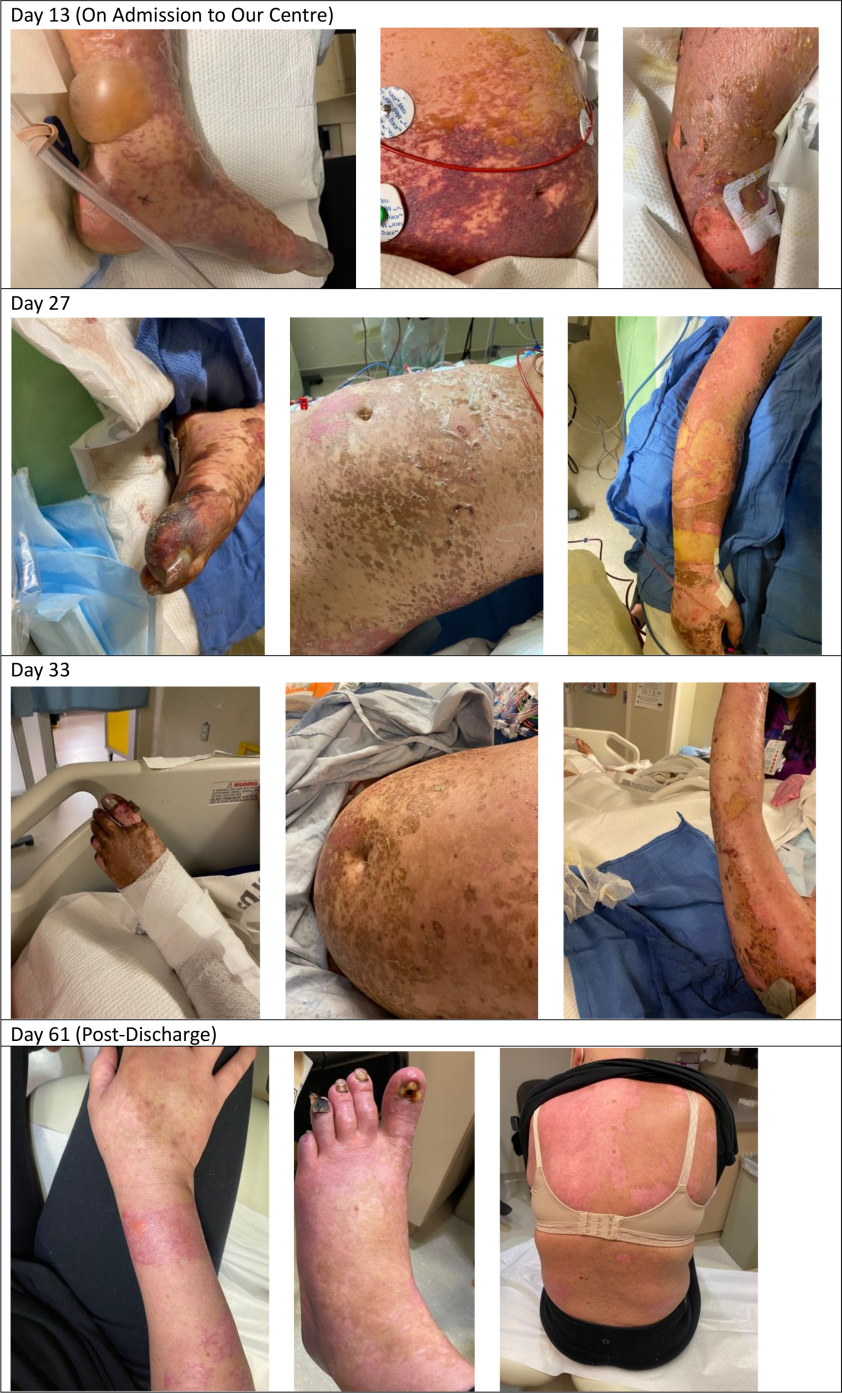
Dermatological toxicities over time.

Eight days later, our patient presented to her local emergency room with fevers. She was found to be neutropenic, admitted to hospital, and treated with antibiotics and acyclovir. She developed an acute kidney injury, with a creatinine that initially increased to 118 µmol/L and rapidly progressed (**Table 1**). This was thought to be secondary to hypovolemia, antibiotics, and antivirals. She received granulocyte-colony stimulating factor (G-CSF) with improvement in her neutrophil count, defervescence, and clinical improvement. However, after 6 days of antibiotics, she developed recurrent fevers over 40°C and became tachycardic. Her antibiotics were broadened, septic workup was repeated, and immune toxicity workup was suggested by medical oncology on call. She then had sudden deterioration ten days into her hospital admission resulting in a pulseless electrical activity arrest, associated with rapidly evolving multiorgan failure including hepatitis, renal failure, respiratory failure, and a rapidly progressive purpuric and tense bullous rash that started peripherally and spread centrally (**Figure 1**). After resuscitation, she was admitted to intensive care where she was intubated, sedated with propofol, and commenced on vasopressor support. A bedside echocardiogram was normal. After consulting with medical oncology, she was started on pulse dose methylprednisolone and, once stabilized, she was transferred to the intensive care unit for continuous renal replacement therapy (CRRT) and ongoing management.

**Table 1:** Laboratory parameters over time.

Lab value	*Reference value*	Admission	PEA arrest day	HLH-2004 D1	HLH-2004 D4	HLH 2004 D8	Pre- Toci dose 1	Pre- Toci dose 2	Post- Toci	Discharge
		Day 0	Day 10	Day 13	Day 16	Day 20	Day 27	Day 28	Day 30	Day 48
**Ferritin**	*4–205 µg/L*			128,619	20267	4573	3213	3627	3949	3237
**LDH**	*120–315 U/L*			5210	559	414		188		359
**CRP**	*0–1 mg/L*		317.1	312.6	98.4	28.0	242.6	267.6	28.3	0.4
**AST**	*10–35 U/L*			1186	64	84	13	8	15	33
**ALT**	*8–40 U/L*	31	29	615	124	131	50	37	40	127
**Cr**	*0–85 umol/L*	91	379	531	211	163	123	128	122	111
**WBC**	*4.0–11.0 x10 9/L*	1.18	6.33	17.5	23.0	5.8	0.5	0.7	6.2	16.0
**Neut**	*2.0–7.50 x 10 9/L*	0.11	2.91	14.13	22.36	4.78	0.02	0.05	3.00	13.87
**Hb**	*115–155 g/L*	86	79	64	78	78	87	84	87	85
**Plt**	*150–450 x10 9/L*	83	101	50	66	64	11	15	34	119

If the lab value of interest was not available for a specified timepoint but a value within 1–2 days was available, this is provided in place.

PEA, pulseless electrical activity; LDH, lactate dehydrogenase; CRP, C-reactive protein; AST, aspartate transaminase; ALT, alanaine transaminase; Cr, creatinine; WBC, white blood cells; Neut, neutrophils; Hb, hemaglobin; Plt, platelets; Toci, tocilizumab.

At the time of her transfer, the differential diagnoses included Stevens-Johnson syndrome (SJS), drug rash with eosinophilia and systemic symptoms (DRESS), and immunotherapy-related toxicity. She was then found to have an elevated ferritin (>120,000) and triglycerides (11), raising the possibility of HLH. Her viral investigations were negative. Dermatology, hematology, medical oncology, infectious diseases, and rheumatology were consulted. Her H-Score demonstrated a 98%–99% probability of HLH, and she was started on the HLH-2004 protocol with etoposide. High dose methylprednisone 1g daily was continued. Bone marrow biopsy did not demonstrate clear cytologic evidence of active phagocytosis, though did demonstrate histiocytes containing residual cellular debris, leading to some disagreement amongst our pathologists regarding whether there was evidence of hemophagocystosis. Skin biopsy demonstrated thrombotic vasculopathy, and dermatology felt that her rash was not secondary to SJS or DRESS. Bullous pemphigoid was also ruled out [direct immunofluorescence (DIF) negative]. The HLH protocol was continued as her ferritin, C-reactive protein (CRP), liver enzymes, and renal function improved, and her respiratory status stabilized. Her methylprednisolone dosing was decreased to 100 mg/day, and then further weaned to 80 mg/day. After the third dose of etoposide, she developed profound pancytopenia, and the fourth dose was held. She was recommenced on G-CSF and was given both platelet and red cell transfusions. Throughout, she required extensive wound care related to the bullous rash, which ultimately resulted in desquamation covering approximately 50% of her body (**Figure 1**).

Unfortunately, 13 days after the initiation of the HLH-2004 protocol, our patient began to clinically worsen. She became febrile, required increased ventilator support, and her CRP began to rise rapidly (from a low of 28 mg/L to a peak of 242 mg/L over 4 days). Cultures were obtained and, while pending, she was treated with tocilizumab due to ongoing consideration of cytokine release syndrome. She received two doses (8 mg/kg), 24h apart. Eventually, one of four blood cultures was positive for *Candida lusitaniae*, and she was started on casopfungin, later narrowed to fluconazole. Her clinical status improved, she had no further fevers, and there was rapid improvement in her CRP which fell from a peak of 267 mg/L to 8 mg/L in the 3 days following tocilizumab.

**Figure 2:**
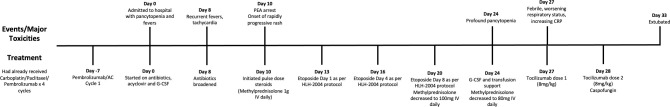
Timeline of events.

She was eventually able to come off CRRT and was extubated 23 days after her initial intubation. She was transferred to the ward, where she continued to receive extensive wound care and was eventually discharged home eight weeks after her initial presentation to hospital. Follow-up CT chest, abdomen and pelvis, and MRI breast have demonstrated a complete radiographic response of the breast cancer and eventual resection showed a complete pathologic response. Complete timeline of the events is found in **Figure 2**.

## Discussion

CRS and HLH have overlapping clinical and biochemical features, which can confound the diagnosis. Immunotherapy has been implicated as a potential cause of both syndromes. Common clinical features include fever, malaise, hypotension, hypoxia, and end organ toxicity. Overlapping biochemical and laboratory findings include elevated creatinine, transaminitis, and elevated inflammatory markers including CRP. Moreover, a CRS-variant with HLH-like manifestations is recognized in patients receiving CAR T cells (12). CRS and HLH are serious complications from immunotherapy, with fatal outcomes occurring in approximately 10% of patients (13).

Although CRS and HLH are more associated with chimeric antigen receptor T-cell therapy and bispecific T-cell engagers, a growing number of published case reports describe CRS and HLH secondary to ICIs (14–32). In a 2020 analysis of the World Health Organization global database of drug-related adverse events, there were 43 reported cases of CRS associated with PD-1/PD-L1 therapy (33). A systematic review of hyperinflammatory syndromes such as CRS and HLH from ICI identified 49 articles and 189 patients and found that pembrolizumab was the most commonly implicated ICI (13). Most case reports have been in the context of metastatic disease, and none have been documented in patients receiving neoadjuvant treatment for breast cancer.

The diagnostic criteria for HLH requires five of eight of the following: fever, splenomegaly, cytopenias affecting greater than two of three lineages, hypertriglyceridemia and/or hypofibrinogenemia, hemophagocytosis in the bone marrow, spleen or lymph nodes in the absence of malignancy, low or no NK cell activity, ferritin greater than 500 µg/L and a soluble IL-2 (sCD25) greater than 2400 U/mL (34). Very high ferritin levels often lead clinicians to consider the diagnosis of HLH, and a ferritin > 10,000 µg/L reportedly has a sensitivity of 90% and a specificity of 96% for macrophage activation syndromes/HLH (35). However, ferritin may also be elevated in the context of sepsis and critical illness, although typically not to the same degree (36). Moreover, CRS may also cause hyperferritinaemia (19); the degree of ferritin elevation appears to correlate with the severity of CRS (37). The elevated triglycerides in this case also increased the H-score. Propofol, which was used for sedation in this patient, can cause elevated triglycerides (38) and could have further confounded the diagnosis. To try and clarify the diagnosis, a soluble CD25 level prior to steroid administration was ordered but not resulted due to limitations on how long samples are held at peripheral hospitals. The uncertainty regarding the presence of hemophagocytosis on the bone marrow further complicated the diagnosis. Although hemophagocytosis is not required for a diagnosis of HLH, it may have aided in distinguishing between HLH and CRS (10, 39).

Despite the clinical, biochemical, and diagnostic overlap of CRS and HLH, the two entities are, in theory, treated differently. HLH is classically treated using the HLH-2004 protocol, which combines IV etoposide and high-dose dexamethasone (40). In contrast, depending on the grade of CRS, tocilizumab and high-dose corticosteroids are the recommended treatments (41). However, in the context of CAR-T–associated HLH, the CAR-T-cell-therapy-associated TOXicity (CARTOX) Working Group have suggested patients be initially managed as per the CRS pathway with anti-IL-6 therapy and high-dose steroids. If there is no improvement after 48h, consideration should then be given to treating with etoposide as per the HLH-2004 protocol (42). Similar treatment sequencing may be beneficial in patients developing HLH/CRS overlap syndromes in the context of ICI.

In reviewing cases of HLH secondary to immunotherapy, we found variability in management. Some patients were treated with the HLH-2004 protocol (22, 27, 31, 43, 44), while others were treated with corticosteroids alone (23, 24, 26, 28, 32, 35, 45–47). Some centers utilized combinations of corticosteroids and other agents such as intravenous immunoglobulin (IVIG) (29, 48), mycophenolate mofetil (MMF) and cyclosporin (30), anakinra (29, 49), tocilizumab (31, 50), and infliximab (25). The management of CRS from immune checkpoint inhibition also varied among published case studies, from high-dose steroids alone (16, 17, 20, 21) to combination therapy with steroids, MMF, plasma exchange, and IVIG (18). Given the diagnostic uncertainty in our case, and given the biochemical and clinical improvement seen after the initiation of the HLH protocol, treatment was continued; however, high-dose steroids were also continued to manage possible CRS. With subsequent deterioration, IL-6–directed therapy was instituted with tocilizumab and, in parallel, antifungals were administered. It remains unclear whether this second improvement was predominantly due to tocilizumab or appropriate management of fungemia.

The cutaneous involvement in this case was unusual. Our patient’s severe bullous rash began approximately 9 days after she initially presented with fevers. In the literature, there are seven case reports describing ICI-induced HLH with an associated rash. Four of the HLH case reports documented a maculopapular rash (22, 23, 43, 49). Sasaki et al. (46) reported pembrolizumab associated HLH with an erythema multiforme-like, full body rash. Choi et al (48) described a pruritic truncal rash that progressed to papules and erosions, and eventually peripheral and oropharyngeal bullae, but skin biopsies from this case were consistent with SJS. There are two documented cases of CRS from ICI with an associated rash. Tsutsui et al. (20) described CRS secondary to ipilimumab and nivolumab with a progressive rash that started on the patient’s neck and spread peripherally with mucosal involvement. A skin biopsy was consistent with toxic epidermal necrolysis. Amlani et al. (15) describe a patient who developed a purpuric eruption on his legs after ICI, which progressed to his entire body.

Our case was also unusual given the curative intent of treatment. Adjuvant or neoadjuvant ICI is currently approved for use in TNBC (7), melanoma (51), Bacillus Calmette-Guerin (BCG) unresponsive non-muscle invasive bladder cancer (52), non-small cell lung cancer (53), renal cell carcinoma (54), esophageal cancer (55), and is being investigated in many other disease sites. Recent positive data using neoadjuvant immunotherapy for patients with estrogen receptor–positive breast cancer, including Keynote-756 (56) and Checkmate-7FL (57), may further expand the number of eligible patients. ICIs in these patient populations will pose unique challenges, including possible exposure of younger, curative intent patients to potentially life-threatening and long-term side effects.

## Conclusion

This is the first case report in the literature of HLH/CRS in a patient with breast cancer being treated with curative intent. As ICI indications expand further into the adjuvant and neoadjuvant population, careful consideration must be given to rare but potentially life-threatening complications in patients receiving curative therapy. Prompt recognition and early collaboration with specialists is vitally important to avoid fatal outcomes.

## Data availability statement

The original contributions presented in the study are included in the article/supplementary material. Further inquiries can be directed to the corresponding author.

## Ethics statement

The studies involving humans were approved by Queen’s Research and Ethics Board. The studies were conducted in accordance with the local legislation and institutional requirements. Written informed consent for publication of this case report was provided by the patient. Written informed consent was obtained from the individual(s) for the publication of any potentially identifiable images or data included in this article.

## Author contributions

LP: Writing – original draft, Methodology, Investigation, Formal analysis, Conceptualization. BM: Writing – review & editing, Methodology, Investigation. PH: Writing – review & editing, Methodology, Investigation. TH: Writing – review & editing, Methodology, Investigation. BW: Writing – review & editing, Supervision, Resources, Methodology, Investigation, Conceptualization.
